# Feasibility Study of Pervious Concrete with Ceramsite as Aggregate Considering Mechanical Properties, Permeability, and Durability

**DOI:** 10.3390/ma16145127

**Published:** 2023-07-20

**Authors:** Shan Gao, Kainan Huang, Wenchao Chu, Wensheng Wang

**Affiliations:** 1 Guangxi Xinfazhan Communication Group Co., Ltd., Nanning 530022, China; liuzyjlu@gmail.com; 2 College of Transportation, Jilin University, Changchun 130025, China; 3 Guangxi Transportation Science and Technology Group Co., Ltd., Nanning 530007, China; 4 China State Construction Railway Investment & Engineering Group Co., Ltd., Beijing 100053, China; luogbjlu@163.com

**Keywords:** ceramsite aggregate, compressive strength, freeze–thaw resistance, permeability, pervious concrete

## Abstract

Concrete with light weight and pervious performance has been widely recognized as an effective and sustainable solution for reducing the negative impacts of urbanization on the environment, as it plays a positive role in urban road drainage, alleviating the urban heat island effect and thermal insulation, as well as seismic performance, etc. This research paper presents a feasibility study of pervious concrete preparation with ceramsite as aggregate. First, pervious concrete specimens with different types of aggregates at various water–cement ratios were prepared, and the mechanical properties of pervious concrete specimens were evaluated based on the compressive strength test. Then, the permeability properties of the pervious concrete specimens with different types of aggregates at various water–cement ratios were characterized. Meanwhile, statistical analysis and regression fitting were conducted. Finally, the analysis of the freeze–thaw durability of pervious concrete specimens with ceramsite as aggregate according to indexes including quality loss rate and strength loss rate was performed. The results show that as the water–cement ratio increased, the compressive strength and permeability coefficient of pervious concrete generally decreased. Compressive strength and permeability coefficient showed a great correlation with the water–cement ratio; the R^2^ values of the models were around 0.94 and 0.9, showing good regression. Compressive strength was mainly provided by the strength of the aggregates, with high-strength clay ceramsite having the highest 28-day compressive strength value, followed by ordinary crushed-stone aggregates and lightweight ceramsite. Porosity was mainly influenced by the particle size and shape of the aggregates. Lightweight ceramsite had the highest permeability coefficient among different types of cement-bound aggregates, followed by high-strength clay ceramsite and ordinary crushed-stone aggregates. The quality and compressive strength of pervious concrete specimens decreased with the increase in freeze–thaw cycles; the quality loss was 1.52%, and the compressive strength loss rate was 6.84% after 25 freeze–thaw cycles. Quadratic polynomial regression analysis was used to quantify the relationship of durability and freeze–thaw cycles, with R^2^ of around 0.98. The results provide valuable insights into the potential applications and benefits of using ceramsite as an aggregate material in pervious concrete for more sustainable and durable infrastructure projects.

## 1. Introduction

Concrete is a widely used building material due to its high compressive strength, durability, and low cost [1,2,3,4]. However, traditional concrete has poor permeability, which leads to issues such as water accumulation, cracking, and spalling [5,6,7,8]. To address this issue, pervious concrete, which has higher porosity and permeability than traditional concrete, has been developed [9,10]. Pervious concrete has been widely recognized as an effective and sustainable solution for reducing the negative impacts of urbanization on the environment. This type of concrete can be used in various applications, such as pavements, sidewalks, and drainage systems, due to its excellent permeability, which allows rainwater to seep through the surface and recharge groundwater resources [11]. However, the mechanical properties, permeability, and durability of pervious concrete are strongly influenced by the choice of aggregate material [12].

Research on the mechanical properties and permeability of pervious concrete has been carried out in the past and has achieved certain results. The strength of pervious concrete mainly comes from the mutual compression among aggregates and the bonding of cementitious materials [13,14]. Therefore, compared with dense concrete, the mechanical properties and durability of pervious concrete are inferior to those of dense concrete. This is also an important factor that restricts the development of pervious concrete. Research started with the preparation process, indicating that compared with the preparation method of vibration molding, pervious concrete formed using compression and mechanical vibration has better mechanical properties [15,16]. Zhong et al. optimized the performance of pervious concrete from the perspective of mix proportions, improved the mix-proportion design of pervious concrete by referring to the design method of ultra-high-performance concrete, and developed high-strength pervious concrete [17]. Li started with the mixing process and explored the impact of different mixing processes on the basic mechanics and permeability of pervious concrete, thus providing a reasonable vibration mixing process [18]. Pieralis et al. predicted the accurate permeability coefficient by simulating the stacking method of pervious concrete and combining parameters such as aggregate particle size and bone-cement ratio [19]. Santamouris, M., summarized the effects of the external photothermal environment (such as solar radiation, convective heat transfer coefficient, air humidity, temperature) and the material itself (thermal conductivity, heat capacity) on the surface temperature of pervious pavement [20]. Liu et al. found that compared with traditional pervious pavement, capillary-evaporation-enhanced pavement can effectively utilize the capillary effect to transport deeper water to the road surface for evaporation and prolong cooling time [21]. Qin et al. established a calculation model for the evaporation of pervious concrete and found that surface moisture content had a significant impact on the evaporation of pervious pavement, and when the surface moisture of pervious concrete was depleted, its surface temperature was actually higher than that of ordinary concrete [22]. Therefore, many scholars’ research on pervious concrete mainly focused on mechanical properties, mix design, forming technology, permeable performance, and other aspects, and there are some advantages and potential benefits to pervious concrete [23,24,25].

The role of aggregates in concrete’s performance has been studied by many scholars. Aggregates play a crucial role in the performance of concrete; they are granular materials, including coarse and fine aggregates, that directly influence the mechanical properties, durability, and workability of concrete [26]. Firstly, aggregates significantly impact the strength and stability of concrete [27,28]. Coarse aggregates such as crushed stone or gravel determine the compressive strength, tensile strength, and load-bearing capacity of concrete. Proper selection of the size and shape of aggregates enhances these properties [29]. Additionally, different aggregate shapes provide better interlocking, increasing the overall stability of concrete and reducing the risk of shrinkage and cracking [30]. Secondly, aggregates contribute to the durability of concrete [31,32]. The quality and chemical stability of aggregates influence concrete’s resistance to chemical corrosion, freeze–thaw cycles, and alkali–aggregate reactions. High-quality aggregates reduce the risk of deterioration caused by these factors and extend the service life of concrete. Moreover, aggregates affect the workability and formability of concrete. Fine aggregates, typically sand or powdered materials, fill the voids among coarse aggregates, improving the density and flowability of concrete [33]. Controlling the proportion and particle distribution of aggregates allows for adjustments in workability, slump, and processability, making construction and shaping easier [34]. Furthermore, the choice of aggregates is influenced by sustainability considerations [35,36]. There has been increasing research on using recycled or alternative materials as aggregates. For instance, recycled aggregates, slag, and fly ash are widely applied in concrete to reduce resource consumption and environmental impacts [37]. In summary, aggregates play a vital role in concrete. They influence its strength, stability, durability, and workability. Proper selection and utilization of aggregates enhance concrete’s performance to meet the specific requirements of various engineering projects. Ongoing technological advancements and sustainable development goals drive further research and innovation in aggregates for the continuous improvement of concrete materials.

The increasing demand for concrete has brought new requirements for its performance, while environmental and energy-saving requirements have also put forward higher requirements for concrete. The lightweight nature, composite performance, and environmentally friendly development of concrete structures are important directions [38]. Compared with ordinary concrete, lightweight aggregate concrete has properties such as insulation, heat insulation, and sound insulation [39,40,41]. The use of expanded clay as a filler is quite wide in Russia [42,43]. Expanded clay concrete is a perspective structural material because of its light weight, and heat- and sound-insulation properties [44]. Expanded clay can effectively improve the compressive strength and elastic modulus of lightweight concrete [45]. In recent years, ceramsite has emerged as a promising aggregate for pervious concrete due to its light weight, low thermal conductivity, and good water-absorption properties [46,47,48]. Ceramsite has shown great potential as a sustainable and economical alternative to traditional aggregates such as gravel and sand [49]. Wang et al. studied the effect of the moisture content in ceramic particles on the early cracking sensitivity of lightweight aggregate concrete using aggregates such as clay ceramic particles and shale ceramic particles [50]. They found that the cracking sensitivity of lightweight aggregate concrete decreased with the increase in moisture content. Meanwhile, due to the pervious nature of ceramic particles, wetting ceramic particles in advance can also prevent moisture absorption during the concrete mixing process, ensuring the reliability of the water–cement ratio in the mix. Hwang et al. added a tackifier to the preparation of shale ceramsite concrete [51]. Based on the experimental results, it was proposed that after adding a tackifier, the viscosity of the mixture slurry would increase and the floating of the aggregates would be improved. However, when the tackifier was added excessively, the slump loss was significant, affecting the pumping in the project. At the same time, after excessive use of the tackifier, the strength of concrete also slightly decreased. Mortazavi et al. investigated the effect of silica fume on the compressive strength of clay-ceramsite concrete [52]. The test results indicate that the compressive strength of clay-ceramsite concrete was highly sensitive to silica fume. With the increase in silica-fume content, the strength increased significantly, but the change in strength slowed down when the silica-fume content was high. The use of ceramsite as a replacement for these aggregates in pervious concrete could improve the mechanical properties, permeability, and durability of the resulting material.

This feasibility study aimed to investigate the use of ceramsite as an aggregate material in pervious concrete and evaluate its mechanical properties, permeability, and durability. The study involved laboratory experiments to determine the compressive strength, flexural strength, and water-absorption capacity of concrete samples, which allowed us to further explore the influence of the water–cement ratio and aggregate types. The novelty of this study is the statistical analysis of compressive strength, permeability, and frost resistance to quantitatively evaluate the influence of the aggregate type on the performance of pervious concrete. The results of this study provide valuable insights into the potential benefits of and challenges to using ceramsite as an aggregate material in pervious concrete. This information could be used by engineers and construction professionals to design more sustainable and durable infrastructure projects that help mitigate the negative impacts of urbanization on the environment.

## 2. Materials and Methods

### 2.1. Raw Materials

In this study, as the important components of pervious concrete, the cementitious material used was P.O.42.5 ordinary Portland cement, and the properties of this cement are listed in Table 1.

**Figure 1:**
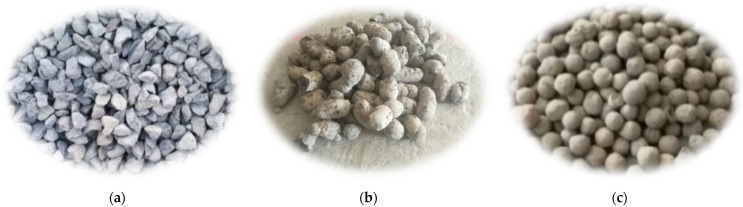
The aggregates used in this study: (**a**) ordinary crushed-stone aggregates; (**b**) lightweight ceramsite; (**c**) high-strength clay ceramsite.

Aggregates are also among the main materials of pervious concrete and are load-bearing materials. The strength of aggregates largely determines the compressive strength of pervious concrete. Therefore, when selecting aggregates, it is important to consider both the permeability and the strength of the aggregates themselves. Different types of aggregates were selected in this study for pervious concrete: one was ordinary basalt crushed-stone aggregates, with a particle size of 9.5 mm, and the other was ceramsite. Ceramsite aggregates included lightweight ceramsite and high-strength clay ceramsite, as shown in Figure 1. The corresponding physical parameters of the above three aggregates are listed in Table 2. The compressive strength of the aggregates was tested according to the Chinese standard “Test methods for natural stones” (GB/T 9966-2020) [53].

In addition, a common method to improve the strength of pervious concrete is to reduce the water–cement ratio by adding a water-reducing agent (WRA). The main admixtures for pervious concrete in this study were high-efficiency naphthalene-series water-reducing agents, with a water-reducing agent dosage of 3% and a water-reducing rate of 10%.

### 2.2. Mix Design and Specimen Preparation of Pervious Concrete

Due to the fact that the preparation of pervious concrete does not include the use of fine aggregates such as sand and gravel, it is not possible to use traditional concrete mixing methods in the mix design process of pervious concrete with cement-bonded aggregates. Therefore, this study used the volume filling theory in the Chinese specification “Technical Specification for Pervious Cement Concrete Pavement” (CJJ/T 135-2009) [54] to determine the performance requirements and proportion recommendations for pervious concrete. The specific dosage of each component was calculated according to the following equation:

*M*_g_/*r*_g_ + *M*_c_/*r*_c_ + *M*_w_/*r*_w_ + *p* = 1, (1)

where *M*_g_, *M*_c_, and *M*_w_ are the amounts of aggregates, cement, and water used per cubic meter of pervious concrete, respectively; *r*_g_, *r*_c_, and *r*_w_ are the densities of aggregates, cement, and water, respectively; and *p* is the porosity of the specimen.

**Figure 2:**
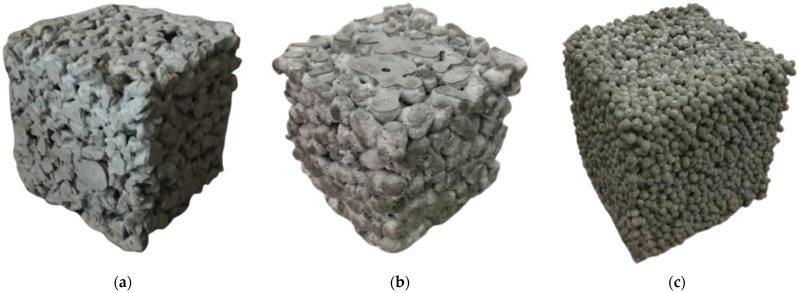
The pervious concrete specimens: (**a**) ordinary crushed-stone aggregates; (**b**) lightweight ceramsite; (**c**) high-strength clay ceramsite.

According to the Chinese specification “Technical Specification for Pervious Cement Concrete Pavement” (CJJ/T 135-2009) [54], the mix design of pervious concrete was carried out. Firstly, the cement dosage for each cubic meter of pervious concrete was set to 200 kg, and a series of gradient water–cement ratios were designed with the water–cement ratio as the design parameter. The water–cement ratios of pervious concrete specimens were designed to be 0.30 with water-reducing agent, 0.30, 0.35, 0.40. The content of other components of pervious concrete was calculated with Equation (1), and the pervious concrete mix is shown in Table 3. The preparation procedure was as follows: For different water–cement ratios and aggregate types, the aggregates and half the water were firstly added into a mixer and stirred for 30 s; then, the rest of the cement was added and mixed for 40 s. Next, the rest of the water was added and stirred for 50 s. The specimens were demolded after 24 h; then, the specimens were cured for 28 days at relative humidity of 95% and temperature of 20 ± 2 °C. Figure 2 shows pervious concrete specimens with the three types of aggregates, including ordinary crushed-stone aggregates, lightweight ceramsite, and high-strength clay ceramsite, respectively.

### 2.3. Experimental Tests

According to the Chinese specification “Standard for Test Method of Mechanical Properties on Ordinary Concrete” (GB/T 50081-2002) [55], the compressive strength test of pervious concrete specimens was conducted using an electro-hydraulic universal testing machine. Before the compressive test, the pervious concrete specimen, and the upper and lower pressure plates of the testing machine were wiped and cleaned, aligning the center of the pervious concrete specimen with the center of the pressure plate. We kept the upper surface of the pervious concrete specimen parallel to the pressure plate before starting the test. During the compressive strength testing process, the testing machine should be kept continuously and stably loaded, with a loading speed of 2 mm/min. When there is a sudden drop in pressure on the testing machine, it should be immediately unloaded, and the maximum pressure at this time is the failure load of pervious concrete. In this study, the average of three tests on pervious concrete specimens was taken for the same concrete mix.

At present, there are two methods for measuring the permeability coefficient of pervious concrete: one is the constant-head testing method, and the other is the variable-head testing method. In this study, according to the Chinese specification “Technical Specification for Pervious Cement Concrete Pavement” (CJJ/T 135-2009) [54], the variable-head testing method was used to design and manufacture a permeability coefficient tester for pervious concrete using organic glass. Permeability is characterized by the water flow rate per unit area of pervious concrete specimens per unit time. Before testing the permeability coefficient, several scales were calibrated on the permeability meter. We placed the permeability meter on the pervious concrete specimens and sealed the connection. Then, about 600 mL of water was poured into the permeability meter, and the water stop valve was quickly removed. Finally, we started timing when the water surface dropped by 100 mL and stopped timing when the water surface dropped by 500 mL to obtain the permeability coefficient.

**Figure 3:**
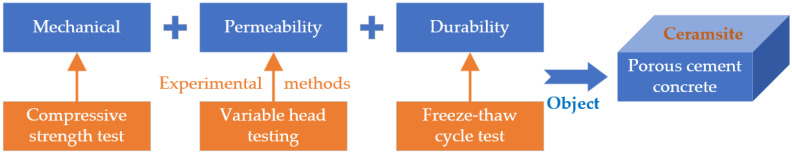
The research diagram of this study.

The pervious concrete specimens were taken out of the curing box after the curing time of 25 d and immersed in water at 20 °C. The water surface had to be 2 cm to 3 cm above the specimen, so that the pervious concrete specimen was fully saturated with water. Three days later, the specimens were taken out of the water and subjected to a freeze–thaw cycle test on pervious concrete. According to the Chinese specification “Standard for Test Methods of Long-term Performance and Durability of Ordinary Concrete” (GB/T 50082-2009) [56], the slow-freezing method was used to study the frost resistance of pervious concrete. The compressive strength and mass of the pervious concrete specimens were measured after 0, 5, 15, and 25 freeze–thaw cycles, respectively. The mass loss rate and compressive strength loss rate are used as evaluation indicators of the freeze–thaw durability of pervious concrete specimens. According to “Standard for Test Methods of Long-term Performance and Durability of Ordinary Concrete” (GB/T 50082-2009) [56], the freeze–thaw cycle test is to be stopped when one of the following three situations occurs: (1) the number of freeze–thaw cycles has been reached; (2) the compressive strength loss rate reaches 25%; or (3) the mass loss rate reaches 5%. Figure 3 shows the research diagram of this study.

## 3. Results and Discussion

### 3.1. Compressive Strength Analysis Considering Water–Cement Ratio and Aggregate Type

**Figure 4:**
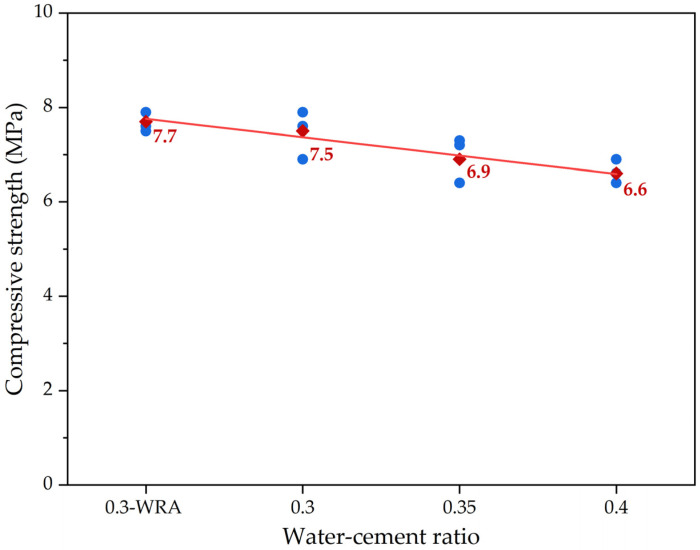
The compressive strength results of pervious concrete with crushed-stone aggregates under different water–cement ratios.

The effects of the water–cement ratio on the 28-day compressive strength of pervious concrete are shown in Figure 4, in which the red square symbols are the average 28-day compressive strength values, and the blue solid circle symbols are the test data. From Figure 4, as the water–cement ratio increased, the compressive strength of pervious concrete with crushed-stone aggregates generally showed a decreasing trend. Because pervious concrete specimens were mainly composed of two components, including aggregates and cement slurry, under the condition that the amount of cement and aggregates, and the aggregate particle size remained unchanged, as the water–cement ratio increased, the amount of water in the pervious concrete specimens gradually increased, resulting in a decrease in the ratio of cement in the cement slurry. The strength of pervious concrete with crushed-stone aggregates mainly depended on the cement slurry layer among the aggregates and on the bonding performance between the cement slurry layer and the aggregates. The aggregate–cement ratio directly determined the thickness and bonding area of the cement slurry layer on the surface of the aggregates, as well as the interlocking effect among the aggregates. From Table 3, it can be seen that for different groups of pervious concrete specimens with different water–cement ratios, the aggregate dosage and particle size of pervious concrete with crushed-stone aggregates remained unchanged. The strength mainly depended on the cement slurry, and as the water–cement ratio increased, the cement slurry layer among the aggregates gradually became thinner. And due to the relatively reduced proportion of cementitious materials, the bonding performance between the cement slurry layer and the aggregates was further weakened. Therefore, as the water–cement ratio increased, the overall compressive strength of pervious concrete with crushed-stone aggregates showed a decreasing trend. Linear regression analysis was used to quantify the relationship between the water–cement ratio and the compressive strength of pervious concrete. It was found that the correlation of parameter water–cement ratio and compressive strength was a great correlation, with R^2^ of the model of around 0.94.

**Figure 5:**
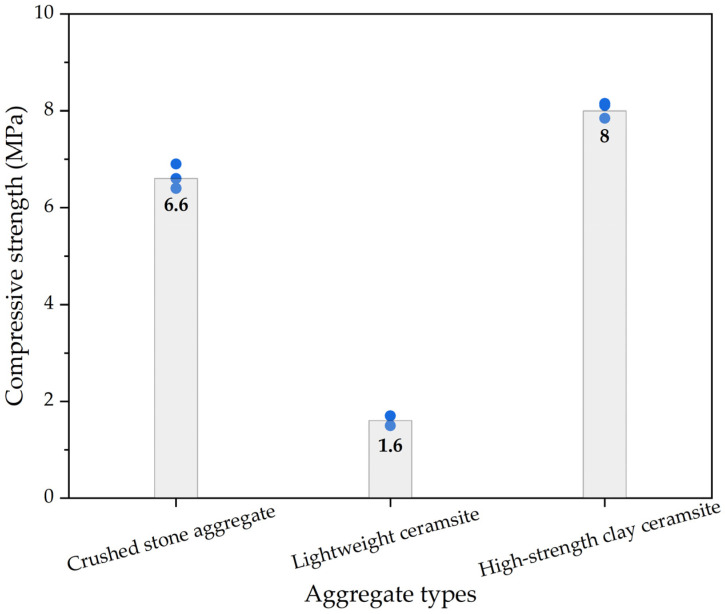
The compressive strength results of pervious concrete with different types of aggregates.

For pervious concrete with different types of cement-bound aggregates, the 28-day compressive strength results of pervious concrete with different types of aggregates obtained with compressive strength tests are shown in Figure 5, in which the columnar bodies are the average 28-day compressive strength values. The compressive strength of pervious concrete was mainly provided by the strength of the aggregates, and the difference in compressive strength of pervious concrete specimens was the difference in the strength of the selected aggregates. In pervious concrete, a portion of cement plays a role in bonding aggregates during the forming process of pervious concrete specimens, and there is also a portion of cement present among the aggregates, providing a certain strength after curing. For pervious concrete with different types of aggregates, the 28-day compressive strength values exhibited the following pattern: high-strength clay ceramsite > ordinary crushed-stone aggregates > lightweight ceramsite. As shown in Table 2, the compressive strength values of aggregates used in the study exhibited the pattern of high-strength clay ceramsite > ordinary crushed-stone aggregates > lightweight ceramsite, which was consistent with the 28-day compressive strength of pervious concrete. Thus, aggregates play an important role in the strength of concrete, which has been reported in previous research [57,58,59].

### 3.2. Permeability Coefficient Analysis Considering Water–Cement Ratio and Aggregate Type

**Figure 6:**
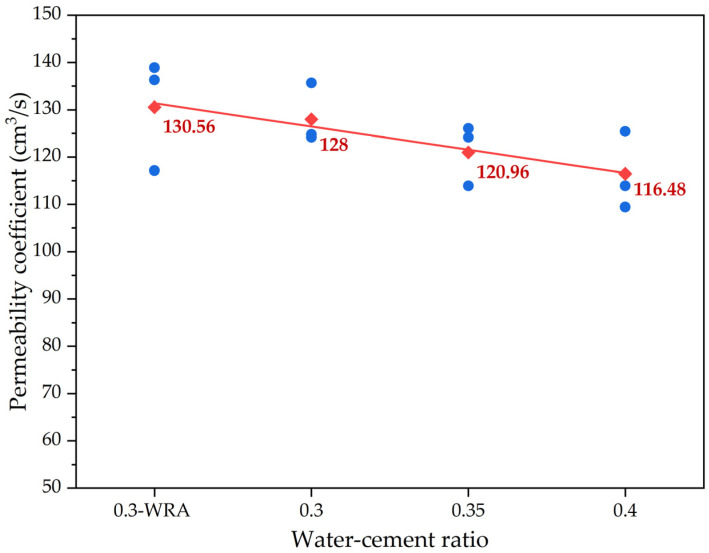
The permeability coefficient results of pervious concrete with crushed-stone aggregates at different water–cement ratios.

Figure 6 shows the effect of the water–cement ratio on the permeability coefficient of pervious concrete; the red square symbols are the average permeability coefficient values, and the blue solid circle symbols are the test data. As the water–cement ratio increased, the permeability coefficient of pervious concrete with crushed-stone aggregates gradually decreased. According to the mix design in Table 3, for different groups of pervious concrete specimens with different water–cement ratios, the aggregate dosage and particle size of pervious concrete with crushed-stone aggregates remained unchanged. As the water–cement ratio increased, the amount of water used in pervious concrete specimens gradually increased, and the volume of cementitious materials increased. This led to a decrease in the porosity of aggregates in pervious concrete specimens, resulting in a decrease in the permeability coefficient. Similarly, linear regression analysis was also used to quantify the relationship between the water–cement ratio and the permeability coefficient of pervious concrete. It was found that the correlation of the parameters water–cement ratio and permeability coefficient was a great correlation, with R^2^ of the model of around 0.96.

**Figure 7:**
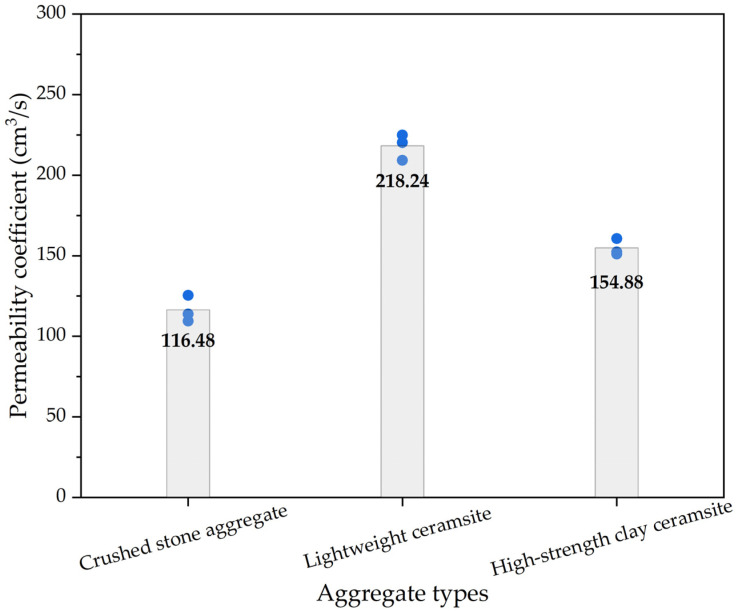
The permeability coefficient results of pervious concrete with different types of aggregates.

For pervious concrete with different types of cement-bound aggregates, the permeability coefficient results are shown in Figure 7, in which the columnar bodies are the average permeability coefficient values. The permeability coefficient of pervious concrete exhibited the following pattern: lightweight ceramsite > high-strength clay ceramsite > ordinary crushed-stone aggregates. This phenomenon was mainly due to porosity, which is influenced by the particle size and shape of the aggregates themselves. Due to the fact that there were only coarse aggregates and no fine aggregates in pervious concrete, there were large pores among the coarse aggregates. The size of the pores is mainly determined by the particle size of the coarse aggregates. The larger the aggregate particle size, the larger the porosity of the pervious concrete specimen composed of coarse aggregates, and the larger the permeability coefficient. As shown in Table 3, the porosity of concrete at the water–cement ratio of 0.40 in the study was sorted as follows: lightweight ceramsite > high-strength clay ceramsite > ordinary crushed-stone aggregates; this was consistent with the permeability coefficient of pervious concrete. It can be seen in Figure 1 that the particle size of lightweight ceramic aggregates was usually large and their shape was irregular. These irregular particles leave some gaps among them, making it easier for water to penetrate into the interior of concrete through these gaps. Meanwhile, compared with other aggregates, lightweight ceramsite concrete had lower compactness. Due to its high porosity and lightweight aggregates, the overall density of concrete was relatively low. This lower density led to more connected pores inside concrete, thereby increasing permeability.

### 3.3. Freeze–Thaw Durability Analysis of Pervious Concrete with Ceramsite

**Figure 8:**
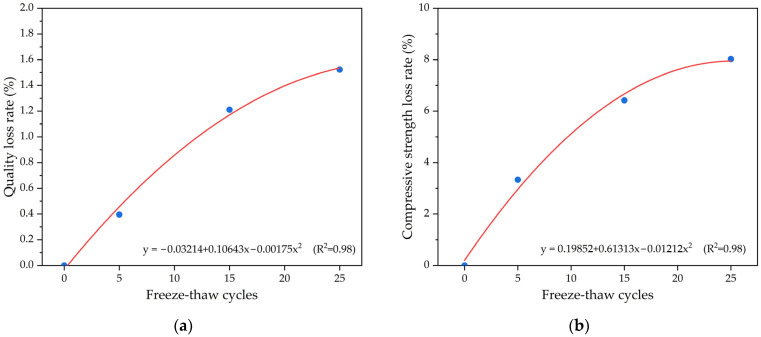
The freeze–thaw durability of pervious concrete specimens with ceramsite: (**a**) quality loss rate; (**b**) compressive strength loss rate.

Freeze–thaw durability, including the quality loss rate and strength loss rate, is plotted in Figure 8 for pervious concrete specimens with ceramsite, respectively. According to Figure 8a, the quality of pervious concrete specimens decreased with the increase in freeze–thaw cycles. The main reason was that during the freeze–thaw cycle of pervious concrete, the free water in the voids condensed into ice, which increased in volume and caused damage stress on the pervious concrete specimens from the inside to the outside. As the number of freeze–thaw cycles increased, this internal destructive tension repeatedly acted between the cementitious material and the aggregates of pervious concrete specimens, leading to a decrease in the bonding force of the cementitious material and the detachment of the aggregates. As a result, the quality of the pervious concrete specimens showed a decreasing trend. When the freeze–thaw cycle had repeated 25 times, the quality loss of the pervious concrete specimens was 1.52%.

**Figure 9:**
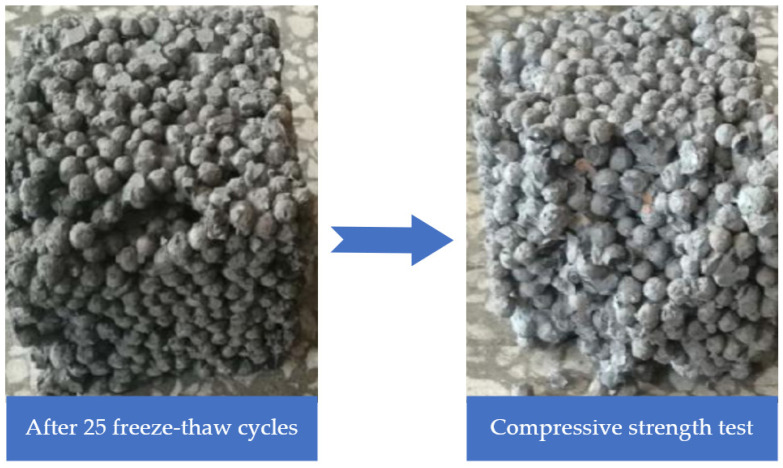
The appearance of a pervious concrete specimen after 25 freeze–thaw cycles.

In Figure 8b, according to the change in the compressive strength loss rate of pervious concrete specimens, it can be seen that the compressive strength of pervious concrete specimens decreased to varying degrees with the increase in freeze–thaw cycles. This was because during the freeze–thaw cycle of pervious concrete specimens, the bonding strength between the cementitious material and the aggregates decreased, and some of the aggregates in the pervious concrete specimens fell off, seriously affecting the overall performance of the pervious concrete specimens, leading to a decrease in the compressive strength of the pervious concrete specimens. When the freeze–thaw cycle had repeated 25 times, the compressive strength loss rate of the pervious concrete specimens was 6.84%. The appearance of a pervious concrete specimen after 25 freeze–thaw cycles is shown in Figure 9, which shows that there was a small area of detachment of ceramic particles on the surface of the pervious concrete specimen. Quadratic polynomial regression analysis was used to quantify the relationship between the freeze–thaw cycles and durability indexes including quality loss rate and compressive strength loss rate of pervious concrete. It was found that the correlation of parameter freeze–thaw cycles and durability loss rate was a great correlation, with R^2^ of the model of around 0.98.

### 3.4. Discussion on Reinforcement of Bonding Surface of Pervious Concrete

**Figure 10:**
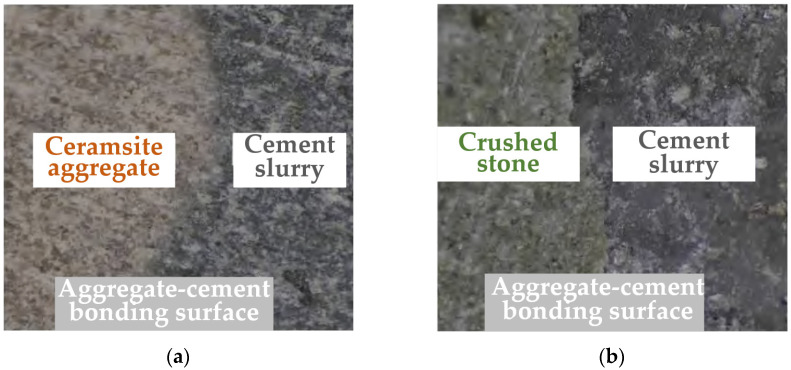
The observation image results of pervious concrete obtained using an optical microscope: (**a**) ceramsite aggregates; (**b**) crushed-stone aggregates.

We observed the bonding surface and physical properties of high-strength ceramic aggregates and crushed-stone aggregates with hardened cement slurry using an optical microscope, and the observation image results are shown in Figure 10. From Figure 10, it is evident that there was a significant difference in the bonding surface between the two types of aggregates and cement slurry. The bonding surface between the crushed-stone aggregates and cement slurry exhibited a clear boundary, whereas the bonding density between the high-strength ceramsite aggregates and cement slurry was good, without a distinct boundary. This disparity can be attributed to the distinct chemical compositions of high-strength ceramic particles and crushed-stone aggregates, leading to variations in their chemical activity. The crushed-stone aggregates had relatively low activity, making it challenging to undergo strong chemical reactions with the cement slurry during the hydration process in the interface transition zone. On the other hand, the high-strength ceramic aggregates, after undergoing high-temperature calcination, exhibited strong surface activity and underwent chemical reactions upon contact with cement, thereby strengthening the contact surface. Consequently, the structure of the interface transition zone became denser. The above analysis and discussion based on the optical microscope results could further explain the mechanisms behind the observed effects of aggregate types on the compressive strength of pervious concrete.

Based on the above research analysis and discussion, it can be inferred that pervious concrete with ceramsite has advantages such as good permeability, environmental protection, and energy conservation and that it can improve soil permeability. Pervious concrete with ceramsite has good permeability, which can effectively reduce rainwater accumulation and the occurrence of water disasters. It can also reduce rainwater runoff, prevent water pollution and the waste of water resources, and meet the requirements for environmental protection and energy conservation. Additionally, it can improve soil permeability, which is beneficial for plant growth and ecological protection. However, the construction requirements of pervious concrete with ceramsite are high, requiring professional construction technology and equipment, and regular maintenance and cleaning to maintain its permeability, which may increase maintenance costs. Thus, applying the results of this research could provide the basis for the possibility of applications in roads and sidewalks, garages and parking lots, parks and gardens, and casing strings for oil wells.

## 4. Conclusions

In this research paper, a feasibility study of pervious concrete preparation with ceramsite as aggregate is discussed. The mechanical and permeability properties of pervious concrete specimens with different types of aggregates at water–cement ratios were investigated. Meanwhile, statistical analysis and regression fitting were conducted. Finally, the analysis of the freeze–thaw durability of pervious concrete with ceramsite as aggregate according to quality loss rate and strength loss rate was performed. Based on the experimental results, the conclusions were drawn:

1. As the water–cement ratio increased, the compressive strength of pervious concrete generally decreased due to higher water content and lower cement ratio in the cement slurry. The thickness and bonding area of the cement slurry layer were directly determined by the aggregate–cement ratio. Compressive strength was mainly provided by the strength of aggregates, with the highest 28-day compressive strength being that of high-strength clay ceramsite, followed by ordinary crushed-stone aggregates and lightweight ceramsite.
2. Pervious concrete’s permeability coefficient decreased with the water–cement ratio due to a decrease in aggregate porosity. Porosity was influenced by aggregate particle size and shape, with larger particles resulting in larger porosity and permeability coefficients. Among different aggregates, lightweight ceramsite had the highest permeability coefficient, followed by high-strength clay ceramsite and ordinary crushed-stone aggregates.
3. The quality of pervious concrete decreased as the freeze–thaw cycles increased, which was caused by internal tension between cementitious material and aggregates. After 25 freeze–thaw cycles, the quality loss was 1.52%. Similarly, compressive strength decreased due to reduced bonding strength between cementitious material and aggregates. After 25 freeze–thaw cycles, the compressive strength loss rate was 6.84%.
4. Linear regression analysis was used to measure the relationship between the water–cement ratio and the compressive strength or permeability coefficient of pervious concrete. The models showed a strong correlation, with R^2^ values of approximately 0.94 and 0.9, indicating a good regression fit. Quadratic polynomial regression analysis was employed to assess the relationship of durability and freeze–thaw cycles, yielding an R^2^ value of around 0.98. After 25 freeze–thaw cycles, pervious concrete specimens exhibited only a small area of detachment of ceramic particles on the surface.

The results of this research could provide the basis for the possibility of applying pervious concrete in roads and sidewalks, garages and parking lots, parks and gardens, and casing strings for oil wells. This study did not consider the influence of cement slurry’s strength, rheological properties, or aggregate-grading characteristics on the mechanics and permeability of pervious concrete, which could be used as further research directions and potential optimization areas.

## Figures and Tables

**Table 1:** The properties of the used cement.

Property	Density (g/cm^3^)	Setting Time (h)		Flexural Strength (MPa)		Compressive Strength (MPa)	
		Initial	Final	7 d	28 d	7 d	28 d
Test	2.9	1	4	4.75	7.65	23.6	43.5
Requirements	-	≥0.75	≤6.5	≥4.0	≥6.5	≥22.0	≥42.5

**Table 2:** The physical parameters of the three aggregates used in this study.

Type	Density (g/cm^3^)	Bulk Density (g/cm^3^)	Crushing Value (%)	Strength (MPa)
Ordinary crushed stone	2.32	1.44	11.3	4.8
Lightweight ceramsite	0.37	0.22	86.4	0.12
High-strength clay ceramsite	1.80	1.10	10.8	5.2

**Table 3:** The pervious concrete mix in this study.

Aggregate Type	Cement (kg)	Water–Cement Ratio	Water (kg)	Aggregate (kg)	Porosity (%)
Ordinary crushed stone	200	0.30	54 (WRA)	1440	25.3
	200	0.30	60	1440	25.1
	200	0.35	70	1440	24.1
	200	0.40	80	1440	23.1
Lightweight ceramsite	200	0.40	80	220	26.1
High-strength clay ceramsite	200	0.40	80	1100	24.1

## Data Availability

Not applicable.
